# IonQuant Enables Accurate and Sensitive Label-Free Quantification With FDR-Controlled Match-Between-Runs

**DOI:** 10.1016/j.mcpro.2021.100077

**Published:** 2021-04-02

**Authors:** Fengchao Yu, Sarah E. Haynes, Alexey I. Nesvizhskii

**Affiliations:** 1 Department of Pathology, University of Michigan, Ann Arbor, Michigan, USA; 2 Department of Computational Medicine and Bioinformatics, University of Michigan, Ann Arbor, Michigan, USA

**Keywords:** proteomics, mass spectrometry, label-free quantification, match-between-runs, false discovery rates, single-cell proteomics, CV, coefficient of variation, DDA, data-dependent acquisition, DIA, data-independent acquisition, FAIMS, high-field asymmetric ion mobility spectrometry, FDR, false discovery rate, LC-MS, liquid chromatography-mass spectrometry, LDA, linear discriminant analysis, LFQ, label-free quantification, MBR, match-between-runs, PSM, peptide-spectrum match

## Abstract

Missing values weaken the power of label-free quantitative proteomic experiments to uncover true quantitative differences between biological samples or experimental conditions. Match-between-runs (MBR) has become a common approach to mitigate the missing value problem, where peptides identified by tandem mass spectra in one run are transferred to another by inference based on m/z, charge state, retention time, and ion mobility when applicable. Though tolerances are used to ensure such transferred identifications are reasonably located and meet certain quality thresholds, little work has been done to evaluate the statistical confidence of MBR. Here, we present a mixture model-based approach to estimate the false discovery rate (FDR) of peptide and protein identification transfer, which we implement in the label-free quantification tool IonQuant. Using several benchmarking datasets generated on both Orbitrap and timsTOF mass spectrometers, we demonstrate superior performance of IonQuant with FDR-controlled MBR compared with MaxQuant (19–38 times faster; 6–18% more proteins quantified and with comparable or better accuracy). We further illustrate the performance of IonQuant and highlight the need for FDR-controlled MBR, in two single-cell proteomics experiments, including one acquired with the help of high-field asymmetric ion mobility spectrometry separation. Fully integrated in the FragPipe computational environment, IonQuant with FDR-controlled MBR enables fast and accurate peptide and protein quantification in label-free proteomics experiments.

## Highlights

• A mixture-model approach controls the false discovery rate of match-between-runs.
• The method is implemented in IonQuant.
• Experiments with various data types show high sensitivity and accuracy of IonQuant.

## Graphical Abstract

**Figure:**
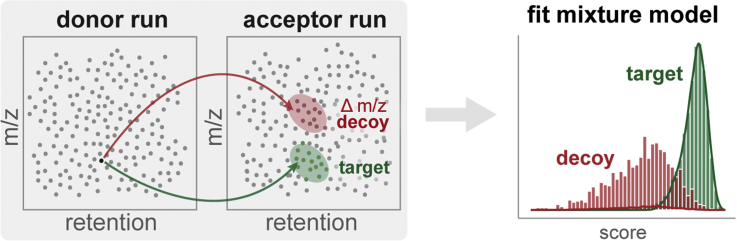

## In Brief

Match-between-runs is a powerful approach to mitigate the missing value problem in label-free quantification. It transfers features identified by MS/MS from one run to the other, but previously, there was no false discovery rate control over this process. We present a mixture model–based approach to estimate and control the false discovery rate, which we have implemented in IonQuant. We demonstrate the sensitivity, accuracy, and speed of IonQuant using proteomics data from timsTOF, Orbitrap, and Orbitrap coupled to FAIMS.

Owing to its sensitive and high-throughput nature, liquid chromatography-mass spectrometry (LC-MS) is a popular technology to identify and quantify peptides and proteins from complex samples. Various approaches to LC-MS data acquisition (1, 2, 3, 4) have been developed, among which data-dependent acquisition (DDA) remains the most commonly used strategy. In the course of a DDA run, eluted peptides are introduced into a mass spectrometer, where peptide ions are sampled for fragmentation and identified from the resulting tandem mass (MS/MS) spectra. Precursor peptide ion intensities are assumed to be correlated with the actual peptide amount, yielding relative peptide and, after an additional peptide to protein roll-up step, protein quantification. Peptide ions successfully targeted and identified by MS/MS are used to calculate peptide and then protein abundances. However, owing to the stochastic nature of intensity-based sampling of peptide ions for MS/MS analysis, not all peptides are consistently identified in all runs. This in turn gives rise to missing quantification values, weakening essential comparisons between different biological samples or experimental conditions. Missing values are generally more prevalent in DDA proteomics than in genomics or transcriptomics. The issue of missing data can be alleviated to some degree using the data-independent acquisition (DIA) strategy (5, 6, 7, 8, 9). However, as label-free quantification using DDA data remains popular, there is a critical need to improve computational solutions for this method.

To address the missing value problem in DDA-based proteomics, a number of “identification transfer” approaches have been devised (10, 11, 12, 13), exemplified by the match-between-runs (MBR) option in MaxQuant (14, 15) that allows “transfer” of identified precursor peptide peaks from one run (referred to below as donor run) to another (acceptor). Given a peak identified by MS/MS in the donor run, attributes, such as m/z, charge state, and retention time, are used to locate a corresponding peak in the acceptor run that is most likely the same peptide. The intensity of the donor peak is then assigned to the acceptor peak, thus filling in the missing value. With more quantified features in common between runs, a greater number of peptides and proteins can be compared among different runs and experiments, increasing the depth of experimental findings (16, 17).

While the goal of MBR is to mitigate the missing value problem, it has the potential to introduce false positives, as transferred peaks have not been rigorously identified using MS/MS spectra in the acceptor run. Lim *et al.* (18) evaluated the false transfer rate of MBR using a two-organism dataset. They concluded that there was a considerable proportion of false positives from MBR when using MaxQuant, yet most were removed with additional filtering as part of the LFQ calculations. However, in practical settings, even with the additional filtering, FDR of MBR may still be unacceptably high. Thus, this subject deserves a more rigorous treatment that can be generalized across different samples and experimental designs. Here, we propose a semisupervised approach to control the FDR of MBR, extending our earlier work on FDR for protein identification (4, 19) and DIA quantification (20, 21). We implement FDR-controlled MBR in IonQuant (22), which has been extended to support LC-MS data both with and without ion mobility. We also implement a new protein abundance calculation module in IonQuant based on the MaxLFQ strategy (15), improving upon our previously described top-N approach (21, 22). Using the dataset from Lim *et al.* (18), we reproduce the authors findings and demonstrate that IonQuant with FDR-controlled MBR has a lower false positive rate and higher sensitivity compared with MaxQuant. With two additional datasets from timsTOF Pro mass spectrometers, we demonstrate that FDR-controlled MBR results in higher quantification precision (lower CV), accuracy, and sensitivity. Finally, we demonstrate that IonQuant displays high sensitivity and precision in single-cell data with and without high-field asymmetric ion mobility spectrometry (FAIMS) separation and that FDR control for MBR is crucial in such datasets. Overall, we propose an efficient approach to perform MBR with FDR control while maintaining high quantification accuracy and precision. We implement the new methods as a default option in IonQuant, readily available as a standalone tool or within our integrated computational platform FragPipe (https://fragpipe.nesvilab.org/).

## Experimental Procedures

### Experimental Design and Statistical Rationale

We used five datasets in this work. In all datasets, we estimated the identification false-discovery rate using the target-decoy approach (4). For MSFragger, peptide-spectrum matches (PSMs), peptides, and proteins were filtered at 1% PSM and 1% protein identification FDR. For MaxQuant, PSMs and peptides were filtered at 1% PSM FDR, and proteins were filtered at 1% protein FDR, which is MaxQuant’s default setting. A two-organism dataset (*H. sapiens* and *S. cerevisiae*) with 40 LC-MS runs from Lim *et al.* (18) was generated on an Orbitrap Fusion Lumos mass spectrometer (Thermo Fisher Scientific). In this dataset, 20 runs include only *H. sapiens* proteins, whereas the remaining 20 runs contain a mixture of *H. sapiens* and *S. cerevisiae* proteomes. *S. cerevisiae* peptides transferred to the 20 *H. sapiens*-only runs by MBR are false positives and were used to evaluate the false positive rate. We also employed two datasets from timsTOF Pro (Bruker), as in our previous work (22). A HeLa dataset with four replicate injections from Meier *et al.* (23) was used to evaluate the sensitivity (*i.e.*, quantified protein count) and precision (*i.e.*, coefficient of variation [CV]) of quantification across replicate runs. A three-organism timsTOF dataset (*H. sapiens*, *S. cerevisiae*, and *E. coli*) with six runs from Prianichnikov *et al.* (24) was used to evaluate quantification accuracy and contains two experimental conditions with ground truth protein ratios: 1:1 (*H. sapiens*), 2:1 (*S. cerevisiae*), and 1:4 (*E. coli*). A single-cell dataset published by Williams *et al.* (25) was generated on an Orbitrap Fusion Lumos mass spectrometer (Thermo Fisher Scientific). This dataset contains three replicate runs with 0 cell (blank runs), 11 replicates with one cell, four replicates with three cells, four replicates with ten cells, and four replicates with 50 cells. Numbers of quantified peptides and proteins were used to evaluate sensitivity, and quantification CV was used to evaluate precision. The last dataset was also from a HeLa single-cell experiment (26), acquired on an Orbitrap Eclipse Tribrid mass spectrometer with the help of FAIMS separation. There are three single HeLa cell runs, three blank runs, and three library runs generated from 100 cells. Numbers of quantified proteins were used to evaluate sensitivity.

### Indexing-Based MBR

We developed a fast MBR algorithm based on indexing. In IonQuant (22), an index of each run is built and written to the disk for fast feature extraction, which supports data with and without ion mobility information. The peak tracing and normalization modules were improved to make it more sensitive and robust compared with the initial release of IonQuant. The new version performs resampling to make the peaks have the same time interval. Then, it performs Savitzky-Golay smoothing (27), finds the boundaries, and subtracts background noise using Skyline’s approach (https://skyline.ms/wiki/home/software/Skyline/page.view?name=tip_peak_calc). In the normalization module, the whole m/z range is now divided into ten bins with the same number of ions, which makes normalization more robust for sparse data or samples with large differences in abundance.

Given a run with possible missing values that will accept ions (acceptor run) and a separate run that will be used to fill these missing values (donor run), correlations between the two runs are calculated using overlapped ions’ retention times, intensities, and ion mobilities if applicable: $\left(o\times r_{1}+o\times r_{2}\right)/2$ or $\left(o\times r_{1}+o\times r_{2}+o\times r_{3}\right)/3$, where *o* is the overlapping ratio (28); *r*_1_, *r*_2_, and *r*_3_ are Spearman’s rank correlation coefficients of retention time, intensity, and ion mobility, respectively. Up to *n* (user-specified “MBR top runs” parameter, 10 by default) donor runs with the highest correlations (which must be greater than user-specified “MBR min correlation” parameter, 0 by default) are selected.

For each ion in every selected donor run, we locate the target region within the acceptor run using an approach similar to FlashLFQ (29). First, pairs of retention times from the corresponding ions are collected and sorted according to the value from the donor run. Using *d*_*i*_ and *a*_*i*_ to denote the retention times of *i*-th pair of ions from the donor and acceptor runs, respectively, we have pairs from (*d*_1_, *a*_1_) to (*d*_*N*_, *a*_*N*_) sorted by *d*_*i*_, where *N* is the number of overlapped ions. Given a donor ion with retention time *t*, we find its position in the sorted pairs satisfying $d_{i}\leq t<d_{i+1}$. Then, we collect all pairs satisfying $d_{i}-\text{τ}\leq d_{j}\leq d_{i}+\text{τ}$, where τ is a predefined tolerance (“MBR RT window” parameter, 1 min by default). With those pairs, we generate a list whose elements are *a*_*j*_ − *d*_*j*_ and calculate the median (*m*) and median absolute deviation (σ) of that list. The possible target range in the retention time dimension is then:

(1) $$\begin{array}{l} \left[d_{i}+m-2\sigma ,d_{i}+m+2\text{σ}\right] \end{array}$$

If ion mobility data are used, we take the same approach to locate the target range in the ion mobility dimension (controlled by the “MBR IM window” parameter, 0.05 by default). The transferred ion’s m/z equals the donor ion’s m/z adjusted by mass calibration error (mass calibration is performed by MSFragger (30)). After locating the target region in m/z, retention time, and ion mobility if applicable, we trace all peaks within the region using our recently described algorithm (22). Two isotope peaks (+1 and +2) are also traced to check the charge state and the isotope distribution. Peak boundaries are allowed to extend beyond the target region’s retention time and ion mobility bounds. Peak tracing is performed rapidly using the index, after which the donor ion’s peptide information is assigned to the traced monoisotopic peak.

IonQuant can automatically detect if the data were acquired using FAIMS. If FAIMS was used, IonQuant builds separate spectral indexes corresponding to each compensation voltage. Then, peak tracing, ion detection, and ion transfer are performed within each compensation voltage.

### MBR False Discovery Rate Estimation

To estimate the rate at which false transfers occur, we adopted a supervised semiparametric mixture model that we previously applied in a number of related applications (19, 20). For each successfully transferred donor ion (*i.e.*, target ion), we try to transfer a decoy ion, created to have the same retention time and ion mobility (if applicable) but with a large m/z shift (31, 32, 33). To generate a decoy, we first shift the m/z by +11 × 1.0005 Th. If there is no traceable peak in that region, we keep decreasing the m/z shift by 1.0005 Th until we successfully trace a peak or until the m/z shift reaches +4 Th.

For all transferred target and decoy ions, we calculate four (without ion mobility) or five (with ion mobility) scores (Table 1). For one of these scores (using the 0/+1/+2 peaks), Kullback–Leibler divergence is used to compare the quality of the traced isotopic distribution to a theoretical one given m/z and charge state, where the Poisson distribution is used as theoretical (34).

**Table 1:** List of individual scores used to compute the composite score for each transferred ion

Score	Explanation
Log10(intensity)	Log-transformed intensity of a traced peak. The intensity can be from an area (without ion mobility) or a volume (with ion mobility).
Log10(KL)	Log-transformed Kullback-Leibler divergence of an experimental isotope distribution and the theoretical isotope distribution. 0, +1, and +2 isotope peaks are used. The absolute value is also square root transformed.
Abs(ppm)	Absolute value of the mass error (in ppm) from a traced peak. The value is also square root transformed.
IM diff	Ion mobility difference between an acceptor ion and its donor ion. The value is also square root transformed.
RT diff	Retention time difference between an acceptor ion and its donor ion. The value is also square root transformed.

We classify all transferred ions (identified with sequence, charge, and modification information) into four types: a target ion that has not been identified by MS/MS in the acceptor run (type 1); a decoy ion that is from an m/z-shifted type 1 ion (type −1); a target ion that has already been identified by MS/MS (type 2); or a decoy ion that is from an m/z-shifted type 2 ion (type −2). Following the strategy we previously used for DIA data (20), we train a linear discriminant analysis (LDA) model using scores from type 2 and −2 ions. From the trained LDA, we calculate a final score for each type 1 and −1 ion:

(2) $$\begin{array}{l} s=\underset{i}{\sum }w_{i}b_{i} \end{array}$$

where *s* is the final score, *w*_*i*_ are the weights from LDA, and *b*_*i*_ are the scores detailed in Table 1. If multiple ions were transferred to one location, the top scoring one is kept.

Using the final scores from type 1 and −1 ions, we estimate a posterior probability of correct identification transfer by fitting a mixture model:

(3) $$\begin{array}{l} f\left(s\right)={\text{π}}_{0}f_{0}\left(s\right)+{\text{π}}_{1}f_{1}\left(s\right) \end{array}$$

where *f*_*0*_ is the distribution of correctly transferred ions, *f*_*1*_ is the distribution of incorrectly transferred ions, ${\text{π}}_{0}$ and ${\text{π}}_{1}$ are the respective priors of false and true transferred ions. We use the expectation-maximization algorithm (20) to estimate the coefficients and distributions in Equation 3.

After fitting the mixture model, we calculate a posterior probability for each transferred ion using

(4) $$\begin{array}{l} p\left(s_{i}\right)=\frac{{\text{π}}_{1}f_{1}\left(s_{i}\right)}{{\text{π}}_{0}f_{0}\left(s_{i}\right)+{\text{π}}_{1}f_{1}\left(s_{i}\right)} \end{array}$$

where *s*_*i*_ is the score of the transferred ion. Then, we calculate an ion-level MBR FDR using the posterior probability (35) of type 1 ions:

(5) $$\begin{array}{l} \hat{FDR}\left(t\right)=\frac{\sum_{s_{i}\geq t}\left(1-p\left(s_{i}\right)\right)}{\sum_{i}1_{s_{i\geq t}}} \end{array}$$

where *t* is a score threshold and $\underset{i}{\sum }1_{s_{i\geq t}}$ is the number of type 1 ions whose score is larger than *t*. We can also calculate peptide- and protein-level FDR for MBR by collapsing ions with the same sequence or protein and using the highest probability entry in the FDR calculation.

### Calculating Protein Intensity Using MaxLFQ Algorithm

Cox *et al.* (15) proposed MaxLFQ algorithm to calculate protein intensity with peptide intensities. It has a high precision (low CV) according to our previous study (22). We implemented it in IonQuant to provide a new (default) option in addition to the top-N approach.

Given a study with *N* experiments (samples) and a protein with *M* quantified peptide ions, for each peptide ion p∈[1,M], we calculate a log-ratio of its intensities between experiments *i* and *j*:

(6) $$\begin{array}{l} r_{i,j}\left(p\right)=\log\frac{I_{i}\left(p\right)}{I_{j}\left(p\right)}=\log\;I_{i}\left(p\right)-\log\;I_{j}\left(p\right) \end{array}$$

where *I*_*i*_ (*p*) is the intensity of peptide ion *p* from *i*-th experiment. If the ion is not quantified in experiment *i* or *j*, we do not calculate the corresponding log ratio. Then, we have a linear relationship among the log-transformed protein intensities and their peptide ion log-ratios:

(7) $$\begin{array}{l} x_{i}-x_{j}=m_{i,j} \end{array}$$

where *x*_*i*_ is the (unknown) log-transformed protein intensity in *i*-th experiment, and *m*_*i,j*_ is the median of the log-ratios *r*_*i,j*_ (*p*) among all peptide ions *p* from one to *M*. Given the set of one to *N* experiments, Equation 7 can be expressed in a matrix form

(8) $$\begin{array}{l} Ax=b \end{array}$$

where

(9) $$\begin{array}{l} A_{i,j}=\left\{ \begin{array}{l} -1\;\left(i\neq j\right)\\ \overset{N-1}{\underset{i=1}{\sum }}1\left(i,j\right)\;\left(i=j\right) \end{array}\right. \end{array}$$

$$x=\left[\begin{array}{l} x_{1}\\ \vdots \\ x_{N} \end{array}\right]$$

$$b_{i}=\left\{ \begin{array}{l} \overset{N}{\underset{j=i+1}{\sum }}m_{i,j}\;\left(i=1\right)\\ \overset{N}{\underset{j=i+1}{\sum }}m_{i,j}-\overset{i}{\underset{j=1}{\sum }}m_{j,i}\;\left(i>1\right) \end{array}\right.$$

In Equation 9, 1 (*i*, *j*) equals one if there is a peptide ion quantified in both experiment *i* and *j*, and 0 otherwise. Equation 8 can be efficiently solved with Cholesky decomposition to get the log-transformed protein intensity *x*_*i*_. Then, the protein intensity in experiment *i* equals $e^{x_{i}}$.

### Validation of the FDR for MBR Approach Using Two-Organism Dataset

We used 40 runs from Lim *et al.* (18) (ProteomeXchange (36) identifier PXD014415) to evaluate the sensitivity and precision of FDR-controlled MBR. This dataset contains 20 runs with only *H. sapiens* proteins and 20 with a mixture of *H. sapiens* (90%) and *S. cerevisiae* (10%) proteins, all acquired on an Orbitrap Fusion Lumos mass spectrometer. Further sample preparation and data acquisition details can be found in the original publication (18). We used FragPipe (version 13.0) with MSFragger (37) (version 3.0), Philosopher (38) (version 3.2.7), and IonQuant (22) (version 1.5.5) to analyze this dataset. For this analysis pipeline, raw spectral files were first converted to mzML using ProteoWizard (version 3.0.20066) with vendor’s peak picking. We used MaxQuant (39) (version 1.6.14.0) and also Skyline (40) [version Skyline-daily (64 bit) 20.2.1.315 (3785d2eb9)] for comparison. We used raw spectral files for MaxQuant and spectral files converted to the mzML format for other tools. A protein sequence database of reviewed *H. sapiens* (UP000005640) and *S. cerevisiae* (UP000002311) from UniProt (41) (reviewed sequences only; downloaded on Jan. 15, 2020) and common contaminant proteins (26,448 proteins total) was used. For the MSFragger analysis, precursor and (initial) fragment mass tolerance were set to 50 ppm and 20 ppm, respectively. Reversed protein sequences were appended to the original database as decoys. Mass calibration and parameter optimization were enabled. The isotope error was set to 0/1/2, and one missed trypsin cleavage was allowed. The peptide length was set from 7 to 50, and the peptide mass was set to 500 to 5000 Da. Oxidation of methionine and acetylation of protein N termini were set as variable modifications. Carbamidomethylation of cysteine was set as a fixed modification. The maximum allowed variable modifications per peptide was set to 3. Philosopher (38) with PeptideProphet (42) and ProteinProphet (43) was used to estimate the identification FDR. The PSMs were filtered at 1% PSM and 1% protein identification FDR. Quantification and MBR was performed with IonQuant. The minimum number of ions parameter required for quantifying a protein was set to 2 (default). To test the performance of FDR control for MBR, the maximum number of runs used for transfer was set to 40, and the minimum required correlation between the donor and acceptor run was set to 0. Ion-, peptide-, and protein-level MBR FDR thresholds were all set to 1% unless otherwise noted. Protein intensities were computed using the re-implementation of MaxLFQ protein intensity calculation algorithm described above. Default values were used for all the remaining parameters. For MaxQuant comparisons, the parameters were set as close to those described above as possible, with maximum modifications per peptide set to 3, maximum missed cleavages set to 1, LFQ enabled with default settings, maximum peptide mass set to 5000, built-in contaminant proteins were not used, and the second peptide option was not used. Default values were used for all the remaining MaxQuant parameters.

For Skyline comparisons, pep.xml files from PeptideProphet were loaded with a probability threshold 0.9486 that corresponds to 1% peptide-ion level FDR in this dataset. A protein FASTA file filtered with 1% protein FDR was also loaded to make sure that Skyline was processing the peptides additionally filtered with 1% protein FDR. Retention time filtering tolerance was set to 0.4 min, the same tolerance as in IonQuant. After loading all PSMs, we let Skyline generate decoys by reversing the sequences and shifting the precursor masses. Then, we reintegrated the peaks by training a model with the built-in mProphet (44). Finally, we exported a peptide quantification report with estimated q-values, and filtered the data using a 0.01 threshold.

We classified a peptide as an *S. cerevisiae* peptide if it only maps to *S. cerevisiae* proteins. We classified a peptide as *H. sapiens* if it maps to at least one *H. sapiens* protein. The classification was done based on the protein name in the searched protein sequence database: those ending with “_HUMAN” were classified as *H. sapiens* proteins, and those ending with “_YEAST” were classified as *S. cerevisiae* proteins.

### Quantification Precision Comparison Using Four HeLa Cell Lysate Replicates

We used four replicate HeLa cell lysate runs acquired on a timsTOF Pro mass spectrometer (23) with 100 ms TIMS accumulation time to evaluate quantification precision when MBR is used. As in the previous section, we used FragPipe (version 13.0) with MSFragger (version 3.0), Philosopher (version 3.2.7), and IonQuant (version 1.5.5) to analyze this dataset. MaxQuant (version 1.6.14.0) was used to perform a benchmark comparison. Raw spectral files (.d extension) were used. The sequence database contained reviewed *H. sapiens* (UP000005640) proteins and common contaminants from UniProt (downloaded on September 30, 2019; 20,463 sequences). The minimum number of ions parameter required for quantifying a protein was set to 2 unless otherwise noted. For MBR in IonQuant, MBR top runs parameter was set to 3, and MBR min correlation was set to 0. Ion-, peptide-, and protein-level MBR FDR threshold were set to 1%. The remaining parameters were identical to those in the previous section. We used the number of proteins quantified in at least two runs and quantification CV across replicates to evaluate the performance.

### Quantification Accuracy Comparison Using the Three-Organism Dataset

We used the three-organism dataset by Prianichnikov *et al.* (24) to demonstrate the accuracy of IonQuant with MBR. There are six runs from two experimental conditions (A and B) in which *H. sapiens*, *S. cerevisiae*, and *E. coli* proteins are mixed at known ratios. The ratios between conditions A and B are 1:1 (*H. sapiens*), 2:1 (*S. cerevisiae*), and 1:4 (*E. coli*). These data were acquired on a timsTOF Pro mass spectrometer, and details of the sample preparation and data generation can be found in the original publication (24). We used FragPipe (version 13.0) with MSFragger (version 3.0), Philosopher (version 3.2.7), and IonQuant (version 1.5.5) to analyze the data. MaxQuant results published by Prianichnikov *et al.* (24) were used as a benchmark comparison. Using the latest MaxQuant (version 1.6.14.0), a reviewed UniProt protein sequence database and parameters closest to those of MSFragger and IonQuant yielded results similar to those in the original publication (supplemental Fig. S1). A combined database of reviewed *H. sapiens* (UP000005640), *S. cerevisiae* (UP000002311), and *E. coli* (UP000000625) sequences from UniProt (30,788 sequences downloaded Apr. 18, 2020) was used. Ion-, peptide-, and protein-level MBR FDR thresholds were set to 1%. The minimum number of ions parameter required for quantifying a protein was set to 2. Allowed missed cleavages was set to 2, and all other parameters were the same as those in the previous section. We used LFQbench (45) to plot the protein quantification results.

### Single-Cell Dataset Analysis

We used 26 runs published by Williams *et al.* (25) to demonstrate IonQuant’s performance with single-cell data. There are three replicates containing 0 cells which served as negative controls, 11 replicates containing one cell, four replicates containing three cells, four replicates containing ten cells, and four replicates containing 50 cells. The data were generated on an Orbitrap Fusion Lumos mass spectrometer (Thermo Fisher Scientific) over a 30 min LC gradient, with MS/MS spectra acquired in the ion trap. Details of the sample preparation and data acquisition can be found in Williams *et al.* (25). The raw data files were converted to mzML format using ProteoWizard (version 3.0.19302) with vendor’s peak picking. We used FragPipe (version 13.0) with MSFragger (version 3.0), Philosopher (version 3.2.7), and IonQuant (version 1.5.5) to analyze the data. We also used MaxQuant (version 1.6.14.0) as a benchmark. The database was downloaded along with the data (20,129 proteins, ProteomeXchange (36) identifier MSV000085230). In MSFragger analysis, common contaminants and reversed protein sequences were appended by Philosopher. In MaxQuant analysis, the built-in contaminant sequences were used. The precursor mass tolerance was set to 20 ppm, and the initial fragment mass tolerance was set to 0.6 Da. Two missed cleavages were allowed. IonQuant (version 1.5.5) with and without MBR was used. The MBR top runs parameter for MBR transfer was set to 26, and the minimum required correlation was kept at 0. The MaxLFQ protein intensity calculation algorithm was used. The minimum number of ions parameter required for quantifying a protein was set to 1. Multiple ion-level MBR FDR thresholds were applied. The rest of the parameters are the same as those used in the previous section. MaxQuant’s parameters were set as close as possible to those used in MSFragger and IonQuant. We used the numbers of quantified peptides and proteins to evaluate the sensitivity, and we used CV to evaluate the precision of label free quantification with MBR.

### Single-Cell FAIMS Dataset Analysis

We used nine runs published by Cong *et al.* (26) to demonstrate the performance of analyzing single-cell data from an Orbitrap Eclipse Tribrid mass spectrometer (Thermo Fisher Scientific) coupled with FAIMS. There are three single HeLa cell runs, three blank runs serving as negative controls, and three runs with 100 HeLa cells that served as a library for MBR. Each run has two compensation voltages: −55 V and −70 V. The sequence database contains reviewed *H. sapiens* (UP000005640) proteins and common contaminants from UniProt (downloaded on Sep. 30, 2019; 20,463 sequences). We used FragPipe (version 13.0) with MSFragger (version 3.0), Philosopher (version 3.2.7), and IonQuant (version 1.5.5) to analyze the data. Raw spectral files were first converted to the mzML format using ProteoWizard (version 3.0.20253) with vendor’s peak picking. The number of allowed donor runs was set to 9. The rest of the parameters are the same as those used in the previous section. MaxQuant (version 1.6.14.0) was used for comparison. Since MaxQuant does not support FAIMS data natively, we split each raw file into separate mzXML files using FAIMS-MzXML-Generator (https://github.com/PNNL-Comp-Mass-Spec/FAIMS-MzXML-Generator). Scans in each mzXML file have the same compensation voltage (46). Then, we assign fraction number one to the mzXML files with compensation voltage equal to −55 V, and fraction number three to the mzXML files with compensation voltage equal to −70 V (supplemental Fig. S3). In this way, ions are only allowed to be transferred among the files with the same compensation voltage. The rest of the parameters were set as close as possible to those used in MSFragger and IonQuant. We compared the number of quantified proteins with and without MBR from MaxQuant and IonQuant.

### Run Time Comparison

We used the two-organism dataset with 40 Orbitrap Fusion Lumos runs and the HeLa dataset with four timsTOF Pro runs to demonstrate the speed of label-free quantification coupled with FDR-controlled MBR in IonQuant (version 1.5.5). MaxQuant (version 1.6.14.0) was used for comparison. For the two-organism dataset, we used a combined database of reviewed *H. sapiens* (UP000005640) and *S. cerevisiae* (UP000002311) sequences from UniProt (41) plus common contaminants (26,448 proteins downloaded Jan. 15, 2020). For the HeLa dataset, a database of reviewed *H. sapiens* (UP000005640) proteins from UniProt (20,463 proteins downloaded on Sep. 30, 2019) and common contaminants was used. Reversed proteins sequences were appended to both databases as decoys for MSFragger analysis. All other parameters are identical to those used in the previous section. All analyses were run on a desktop with four CPU cores (Intel Xeon E5-1620 v3, 3.5 GHz, eight logical cores) and 128 GB memory. We isolated quantification-specific run times from MaxQuant log files.

## Results and Discussion

### FDR-Controlled MBR

**Fig. 1:**
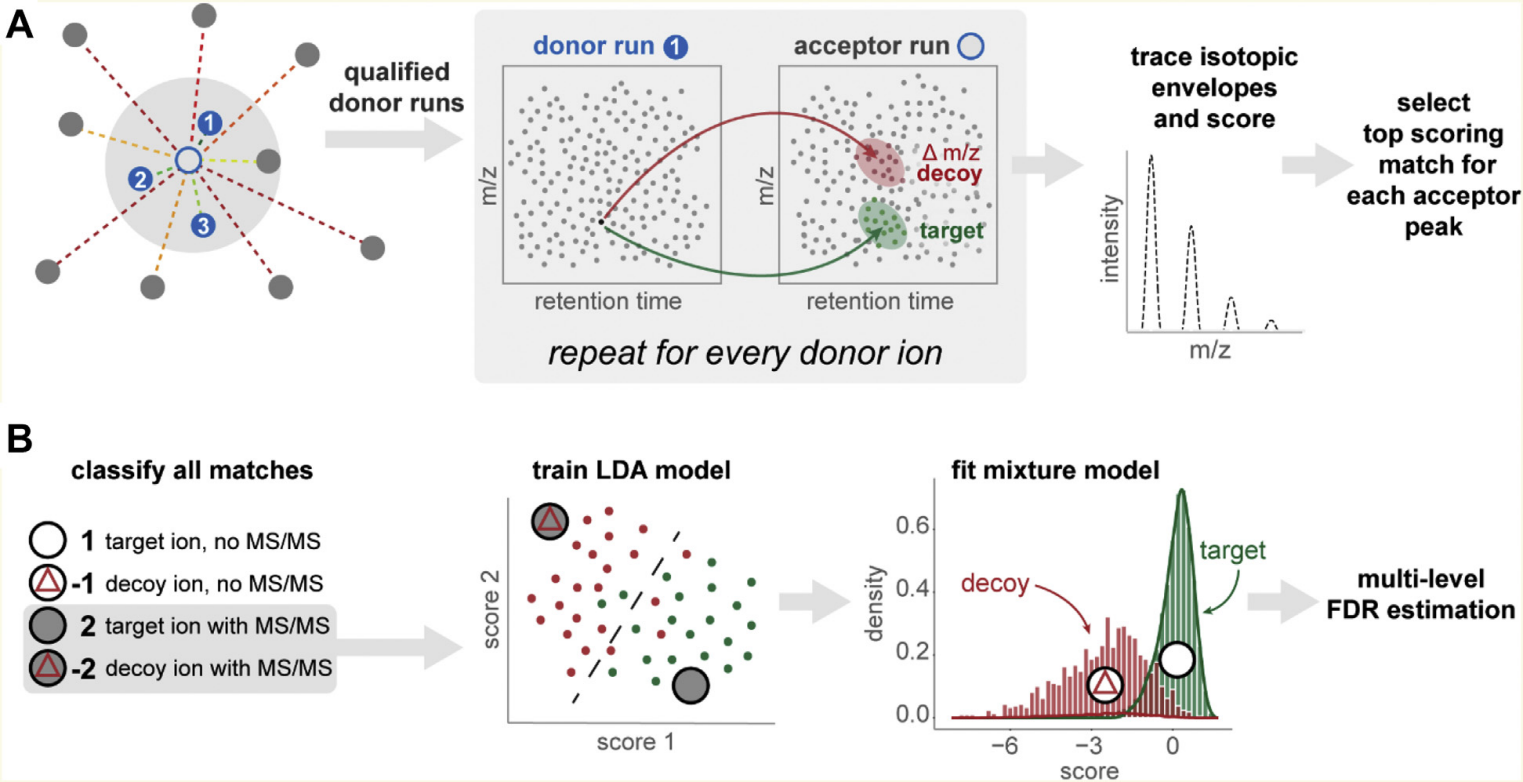
**Overview of match-between-runs in IonQuant.***A,* for each acceptor run (unfilled central point with *blue outline*) ion-level correlations with all other runs (*filled blue* and *gray points*) are calculated, where distance from the central point represents correlation. The top N runs (*numbered blue points*) within the correlation threshold (*gray area*) are selected as eligible donor runs. For every ion in each eligible donor run, target and decoy (m/z-shifted) transfer regions are located using retention time (and ion mobility if applicable). Peak tracing in the acceptor run is used to determine the isotopic distribution and the charge state. All matches are evaluated, and the top scoring donor for each acceptor peak is selected for transfer. *B*, all matches/transferred ions are classified into one of the four categories shown. Type 2 and −2 matches are used to train a linear discriminant analysis (LDA) model. The trained LDA is then used to calculate the final score for type 1 and −1 matches. A posterior probability of correct transfer is estimated by fitting a mixture model, allowing estimation of ion-, peptide-, and protein-level false discovery rate (FDR) for match-between-runs.

We developed an MBR module in IonQuant enabling accurate and fast label-free quantification with match-between-runs peptide ion transfer with the help of the indexing functionality in IonQuant (see Fig. 1 for an overview). For each experiment (acceptor run) in the analysis, ion-level Spearman’s rank correlation coefficients with all other experiments are calculated, where an ion is defined as the combination of peptide sequence, modification pattern, and charge state. The percentage of ions overlapping between two runs is used as a weight in the calculation (28). For each acceptor run, IonQuant picks the top N runs with a correlation larger than a certain threshold as donor runs. Both parameters (“MBR top runs” and “MBR min correlation” can be adjusted by the user). Given an ion from a donor run, IonQuant locates a region in the acceptor run where the transferred ion is likely to be using m/z, retention time, and ion mobility (if applicable) distributions from both runs (see Fig. 1 and Experimental Procedures). For simplicity, we use retention time to describe the region-finding process. Given an ion from a donor run, all ions within a predefined retention time tolerance are collected. Retention time differences from pairs of ions overlapping between the runs are calculated, and the median and median absolute deviation of these differences are found. Then, the region for transfer is determined using Equation 1. We use the same approach to locate the ion mobility region. After getting a 1-D (without ion mobility) or 2-D (with ion mobility) region, IonQuant traces peaks using the donor ion’s m/z, taking any mass calibration correction into account. In addition to the monoisotopic peak, two additional isotope peaks (+1 and +2) are also included in peak tracing so that the isotopic distribution and charge state can be used in the evaluation. Finally, IonQuant assigns the donor ion’s peptide to each traced peak and calculates four (without ion mobility) or five (with ion mobility) scores (Table 1) measuring the quality of the peptide ion transfer.

In conventional MBR, most notably in MaxQuant, ions matching tolerance criteria are transferred without statistically assessing the confidence in the transfer. Here, we propose a semiparametric mixture-modeling approach to estimate the FDR of transferred ions (see Experimental Procedures). Briefly, decoy ion transfers are generated by transferring ions with an m/z shift. All transferred ions are classified into four types: the ion has not been identified by MS/MS (type 1); the ion is a decoy type 1 ion (type −1); the ion has been identified by MS/MS (type 2); and the ion is a decoy type 2 ion (type −2). IonQuant trains a LDA model with type 2 and −2 ions to separate the target and decoy ions. Using the trained model, a final score is calculated for each of the type 1 and −1 ions (Equation 2). A mixture model (Equation 3) is built using type 1 and −1 ions, and the expectation-maximization algorithm is used to fit the model and subsequently calculate the posterior probability. Finally, global ion-level FDR (Equation 5) is calculated using the local FDR, equal to one minus the posterior probability (Equation 4). IonQuant also calculates peptide and protein level FDR by collapsing ions with the same peptide and protein, respectively.

In the remainder of the manuscript, we demonstrate the accuracy of FDR-controlled MBR using a two-organism dataset, and the precision and accuracy of subsequent label-free quantification by using HeLa replicate runs, a three-organism dataset, and two single-cell dataset, respectively.

### Evaluation of FDR-Controlled MBR Method

We used the dataset published by Lim *et al.* (18) to evaluate the false positive rate of FDR-controlled MBR (see Experimental Procedures). The dataset is comprised of 20 LC-MS files from *H. sapiens*-only proteins (“H”) and 20 from a mixture of *H. sapiens* (90%) and *S. cerevisiae* (10%) proteins (“HY”). With MBR, *S. cerevisiae* peptides transferred from HY to H runs are known to be false positives and can be used to evaluate the false positive rate, equal to false positives (*S. cerevisiae* peptides in H runs) divided by negatives (*S. cerevisiae* peptides in total). To ensure all *S. cerevisiae* peptides in the HY runs have the chance to be transferred, the number of top runs used in transferring was set to 40 and minimum required correlation was set to 0. In evaluation, a peptide was assigned to *S. cerevisiae* if all proteins it maps to are from *S. cerevisiae* or to *H. sapiens* if at least one of its proteins is from *H. sapiens*.

**Fig. 2:**
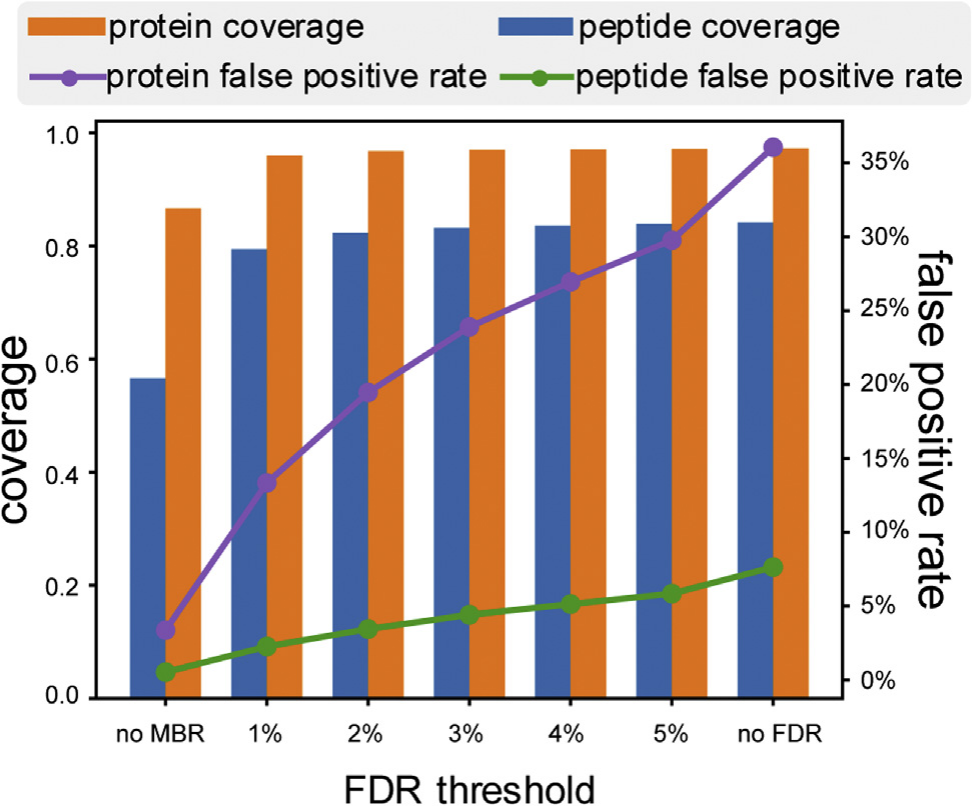
**Per-run proteome coverage and observed false positive rate as a function of the model-estimated false discovery rate (FDR) threshold.** Coverage is equal to the number of *H. sapiens* peptides/proteins from one run divided by the total number of *H. sapiens* peptide/protein identifications in the entire experiment. The false positive rate is equal to the number of *S. cerevisiae* peptides/proteins from one run divided by the total number of *S. cerevisiae* peptides/proteins.

Overall, IonQuant coupled with MSFragger identified 45,875 unique *H. sapiens* peptides and 4610 unique *S. cerevisiae* peptides, ∼19% and ∼31% more *H. sapiens* and *S. cerevisiae* peptides compared with MaxQuant, respectively (Table 2, supplemental Table S1). More peptides were also identified or transferred in individual runs with MSFragger and IonQuant. In transferring ions between the runs, IonQuant had a lower false positive rate than MaxQuant, 2.3% compared with 2.7%. The numbers listed for MaxQuant in Table 2 differ slightly from supplemental Fig. S1 in Lim *et al.* (18) because of small differences in data analysis settings and version of the tools used. Figure 2 shows average peptide coverage, average peptide false positive rate, average protein coverage, and average protein false positive rate with respect to different MBR FDR thresholds. The peptide/protein coverage values shown are *H. sapiens* peptides/proteins in each H run divided by total *H. sapiens* peptides/proteins identified in the dataset. Peptide coverage increases from 57% to 79% with the inclusion of MBR, and protein coverage increases from 87% to 96%. As the MBR FDR threshold is increased, neither peptide nor protein coverage increase significantly, indicating most *H. sapiens* peptides have been successfully transferred by IonQuant already at 1% MBR FDR. The false positive rate continues to rise when the MBR FDR threshold is increased, as expected.

**Table 2:** Peptides quantified by MaxQuant and IonQuant in analyzing the two-organism dataset with MBR

MaxQuant			IonQuant		
Total unique *H. sapiens* peptides	38,405		Total unique *H. sapiens* peptides	45,875	
Sample H, MBR−	19,360 ± 648	50.4%	Sample H, MBR−	26,032 ± 499	56.8%
Sample HY, MBR−	18,945 ± 522	49.3%	Sample HY, MBR−	25,683 ± 716	56.0%
Sample H, MBR+	31,129 ± 637	81.0%	Sample H, MBR+	36,450 ± 283	79.5%
Sample HY, MBR+	29,747 ± 730	77.5%	Sample HY, MBR+	36,113 ± 625	78.7%
Total unique *S. cerevisiae* peptides	3527		Total unique *S. cerevisiae* peptides	4610	
Sample H, MBR−	20 ± 5	0.6%	Sample H, MBR−	26 ± 6	0.6%
Sample HY, MBR−	1848 ± 93	52.4%	Sample HY, MBR−	2597 ± 82	56.3%
Sample H, MBR+	98 ± 10	2.7%	Sample H, MBR+	105 ± 16	2.3%
Sample HY, MBR+	2858 ± 63	81.0%	Sample HY, MBR+	3625 ± 62	78.6%

MSFragger was used to provide identification result for IonQuant. “Sample H” indicates H. sapiens-only samples and “Sample HY” indicates samples with a mixture of H. sapiens and *S. cerevisiae* proteins. There are 20 runs in each sample type. “MBR+” and “MBR-” indicate that the analysis was performed with and without match-between-runs (*MBR*), respectively. For each analysis, unique peptide counts (±range of counts) are listed along with per run identification rates (% of all observed peptides found in each run).

In comparing with the results from Skyline, we noticed that using three scores (intensity, retention time difference, and precursor mass error) had a lower false positive rate (supplemental Table S12), 5.2% *versus* 10.4%, than using the default set of scores in training a model using the built-in mProphet. Despite this improvement, mProphet’s false positive rate remained higher than IonQuant’s (2.3%). The peptide numbers in Skyline without MBR are similar to those from IonQuant because both tools were processing the PSMs from MSFragger.

### Improved Protein Quantification With FDR-Controlled MBR

We used four HeLa cell lysate replicates acquired on a timsTOF Pro published by Meier *et al.* (23) to demonstrate the sensitivity and precision of label-free quantification coupled to FDR-controlled MBR (see Experimental Procedures). We previously (22) performed a similar analysis of the same dataset but without MBR and with protein abundances calculated from peptide ion intensities using top-N peptide approach. In this work, we use a new protein abundance calculation module in IonQuant implemented according to the MaxLFQ (15) algorithm (see Experimental Procedures).

Table 3 lists the numbers of proteins quantified in at least two runs and the median CV from each method. Detailed ion and protein lists can be found in supplemental Tables S2 and S3. The results from IonQuant and MaxQuant (both with MaxLFQ method) are shown, which were run under similar settings of requiring either a minimum of one or two peptide ions in pair-wise ratio calculation in MaxLFQ method (referred to as “Min ions” in IonQuant and “LFQ min. ratio count” in MaxQuant). Enabling MBR (MBR+) improved the number of quantified proteins without a significant increase in protein quantification CV. For example, with min two ion setting, IonQuant MBR+ quantified 9% more proteins (5527 *versus* 5061), while maintaining a CV similar to IonQuant MBR- (medians were 3.6% and 3.5%, respectively). Compared with MaxQuant, IonQuant quantified more proteins and with greater precision (lower CVs) in all pair-wise comparisons between the tools under comparable settings. For example, with minimum ion count set to 1, IonQuant with MBR+ quantified 6346 proteins with a median CV of 4.0%, compared with 5950 proteins with a median CV of 5.3% for MaxQuant with MBR+. IonQuant’s maxLFQ-based protein abundance calculation method also had lower CVs compared with IonQuant with MSstats (47) for peptide to protein intensity roll-up, whereas our initial (top-N peptide based) strategy for protein abundance calculation in IonQuant was inferior to that of MSstats (22) (supplemental Table S13).

**Table 3:** Proteins quantified in at least two runs and median coefficient of variation (CV) from four HeLa cell lysate replicates

Tool			Proteins quantified	Median CV
MaxQuant	MBR−	min 1 peptide	5406	5.3%
		min 2 peptides	4186	4.3%
	MBR+	min 1 peptide	5950	5.3%
		min 2 peptides	5073	4.7%
IonQuant	MBR−	min 1 ion	5971	4.0%
		min 2 ions	5061	3.5%
	MBR+	min 1 ion	6346	4.0%
		min 2 ions	5527	3.6%

“MBR+” and “MBR−” indicate that the analysis was performed with and without match-between-runs (MBR), respectively.

**Fig. 3:**
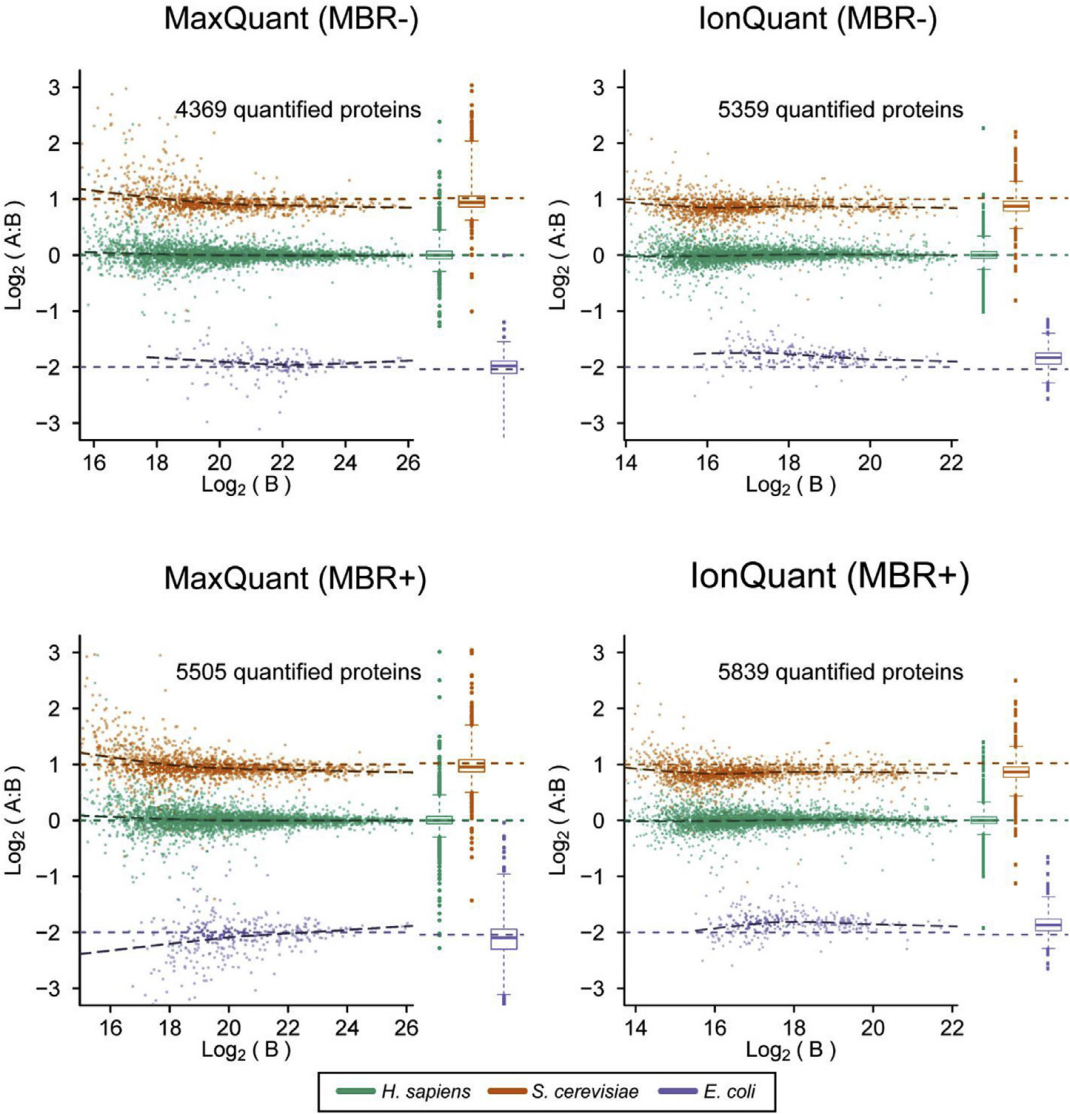
**Ground-truth protein quantification results from MaxQuant and IonQuant from a mixture of three different proteomes.** MaxQuant results are as published by Prianichnikov *et al.* 2020. “MBR+” and “MBR−” indicate that the analysis was performed with and without match-between-runs (MBR), respectively. *S. cerevisiae* proteins are shown in *orange*, *H. sapiens* in *green*, and *E. coli* in *purple*. The known ratios of condition A over condition B are 2:1 (*S. cerevisiae*), 1:1 (*H. sapiens*), and 1:4 (*E. coli*). The *horizontal colored dashed lines* (*orange*, *green*, and *purple*) indicate the true ratios. The *black dashed lines* are fitted curves from observed ratios. *Box plots* of the intensities are shown to the *right* of each scatter plot panel.

We also used the three-organism mixture dataset published by Prianichnikov *et al.* (24) to demonstrate the accuracy of label-free quantification when FDR-controlled MBR is employed (see Experimental Procedures). There are three replicates each of two experimental conditions, where the ratios between the two conditions are 1:1 (*H. sapiens*), 2:1 (*S. cerevisiae*), and 1:4 (*E. coli*). Because these proteomes were mixed at known ratios, we can evaluate the accuracy of the label-free quantification algorithm by comparing the estimated ratio against the ground truth. MaxQuant results published by Prianichnikov *et al.* (24) were used as a benchmark. We also repeated the analysis with a more recent version of MaxQuant (version 1.6.14.0), a newer reviewed protein database, and parameters as close as possible to those used in MSFragger and IonQuant and got similar results (supplemental Fig. S1). We used LFQbench (45) to summarize the analyses and visualize the results (Fig. 3 and supplemental Fig. S2). As expected, both MaxQuant and IonQuant quantified more proteins with MBR than without MBR. IonQuant quantified 6% and 23% more proteins compared with MaxQuant with and without MBR, respectively (Fig. 3, supplemental Tables S4 and S5). IonQuant also had fewer outliers than MaxQuant. The peptide level comparison (supplemental Fig. S2) showed the same trend in comparing IonQuant with MaxQuant.

### FDR-Controlled MBR in Single-Cell Data

We then evaluated the performance of IonQuant with FDR-controlled MBR in single-cell datasets. The first dataset (24) consisted of five biological replicates with 1, 3, 10, and 50 cells. In addition, blank runs (0-cells) were also acquired and used as a negative control for MBR. MaxQuant with and without MBR were used as a benchmark.

**Fig. 4:**
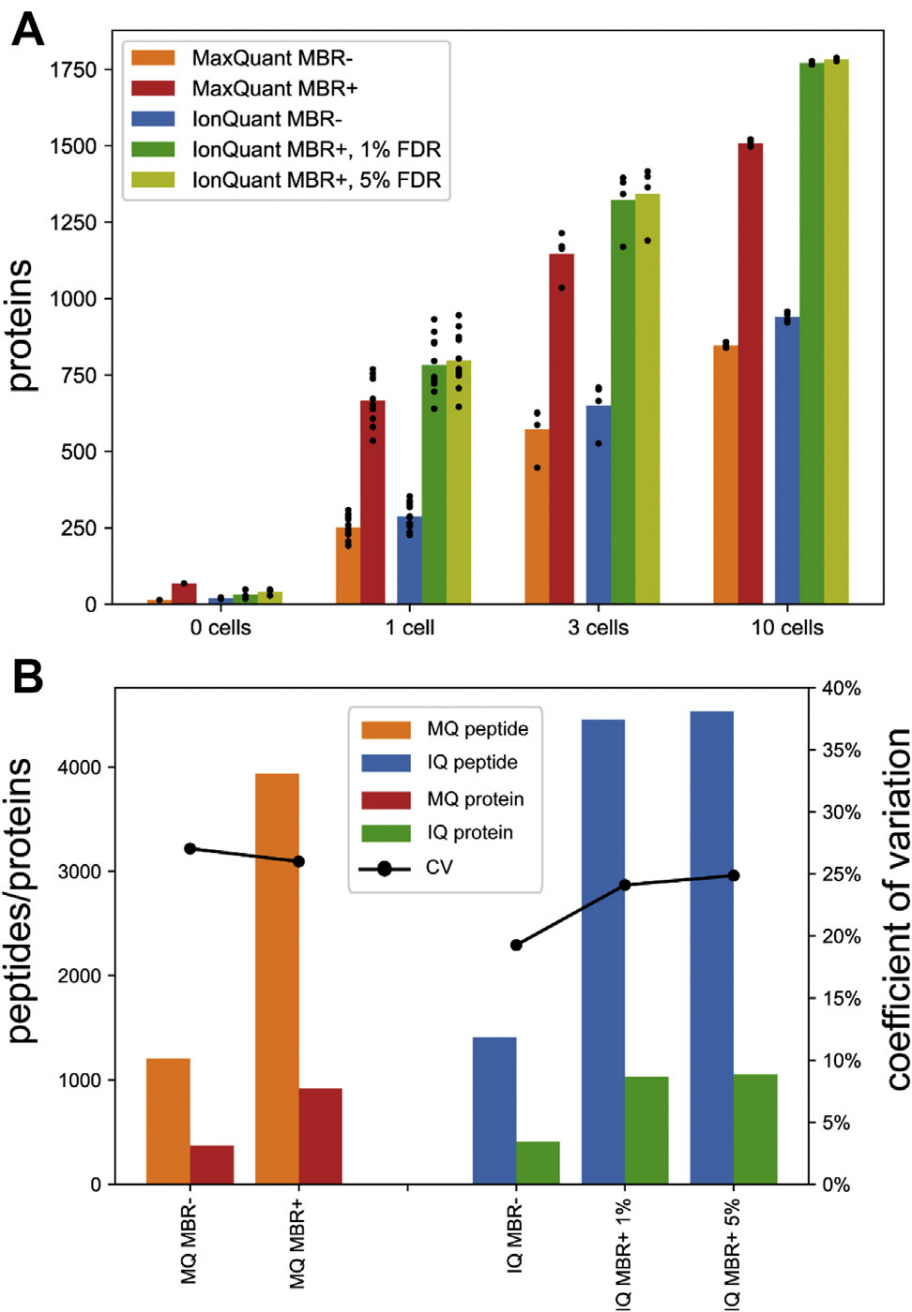
**Peptides and proteins from MaxQuant and IonQuant analysis of the single-cell dataset. “MBR+” and “MBR−” indicate that the analysis was performed with and without match-between-runs, respectively.***A*, numbers of proteins with nonzero intensities from samples with 0 cells (blank runs), one cell, three cells, and ten cells, respectively. Two ion-level MBR false discovery rate (FDR) thresholds (1% and 5%) were applied. *Black dots* indicate the numbers from individual runs. *B*, peptides/proteins quantified in at least two runs and median protein quantification coefficient of variation (CV) from 11 replicates of one cell samples, as a function of FDR threshold. “MQ” indicates MaxQuant and “IQ” indicates IonQuant. *Black curves* and *dots* indicate the median of CV of the corresponding tool.

We first evaluated the number of quantified proteins (proteins with nonzero intensities) (Fig. 4*A*). Detailed ion and protein lists can be found from supplemental Tables S6 and S7. Of note, MaxQuant with MBR (MBR+) reported on average 68 proteins from a replicate of the blank (0-cell) run, which is much more than MaxQuant MBR- (14 proteins), IonQuant MBR- (19 proteins), and IonQuant MBR+ (31 proteins with 1% FDR). This by itself indicates a noticeable false transfer rate of MaxQuant’s MBR in these data. MSFragger with IonQuant, without MBR (MBR-), identified and quantified a higher number of proteins per sample on average than MaxQuant across all groups of samples. As expected, as the number of cells per sample increases, the average number of proteins quantified per sample, with and without MBR, increases for both MaxQuant and IonQuant. Comparing the numbers from MaxQuant MBR+ and IonQuant MBR+ with FDR set to 1% shows that IonQuant still has a higher number of transferred proteins than MaxQuant, which demonstrates the high sensitivity of IonQuant coupled with MSFragger.

Figure 4*B* shows the number of peptides and proteins quantified in at least two runs, and median protein quantification CV from analyzing 11 replicates of 1-cell sample with MaxQuant and IonQuant, respectively. Without MBR, IonQuant measured more peptides (1409 *versus* 1208) and more proteins (406 *versus* 371), while achieving a lower median CV (19.3% *versus* 27.0%) compared with MaxQuant. With MBR and 1% FDR control, IonQuant also measured more peptides (4457 *versus* 3937) and more proteins (1030 *versus* 918) while maintaining a lower median CV (24.1% *versus* 26.0%) compared with MaxQuant.

### FDR-Controlled MBR in Single-Cell Data With FAIMS

We used nine runs (26) from an Orbitrap Eclipse Tribrid mass spectrometer (Thermo Fisher Scientific) coupled with FAIMS to further demonstrate the necessity of controlling FDR for MBR in sparse datasets. There are three blank samples containing cell-free supernatant analyzed as negative control, three single HeLa cell samples, and three samples with 100 HeLa cells to be used as a library for MBR. Each run has two compensation voltages: −55 V and −70 V. MaxQuant with and without MBR was again used for comparison. Because MaxQuant does not natively support FAIMS data, we split each run into two: one has scans with −55 V and the other has scans with −70 V. In MaxQuant analysis, files with different compensation voltages were assigned to different fractions (*i.e.*, 1 and 3, supplemental Fig. S3). IonQuant automatically detects and handles FAIMS data, so this manual step is not necessary.

Table 4 shows the number of quantified proteins (proteins with nonzero intensities) from blank and single-cell HeLa samples (the corresponding ions and protein lists can be found in supplemental Tables S8 and S9). Both MaxQuant and IonQuant with MBR identified a relatively large number of proteins in the blank samples (79 and 97 on average per replicate, respectively). This suggests that the blank samples in this experiment cannot be considered as true negative controls for MBR, further highlighting the need for statistical FDR control. While MaxQuant with MBR+ quantified significantly more proteins in the single-cell samples than with MBR- (on average, 1230 *versus* 557), with MBR+, it also reported on average 492 proteins in the blank samples. In contrast, IonQuant with MBR+ and 1% FDR quantified a comparable number of proteins (on average, 1156) in the single-cell runs as MaxQuant with MBR+; however, the number of quantified proteins in the blank samples has not increased as significantly as with MaxQuant. Applying more lenient MBR FDR thresholds of 2% or 5% in IonQuant results in a significant increase in the number of quantified proteins, whereas the number of proteins in the blank samples increases as well but still stays below that of MaxQuant with MBR+.

**Table 4:** Number of proteins with nonzero intensities from MaxQuant (MQ) and IonQuant (IQ)

Data type	MQ MBR−	MQ MBR+	IQ MBR−	IQ MBR+, 1% FDR	IQ MBR+, 2% FDR	IQ MBR+, 5% FDR
Blank	79 (152)	492 (887)	97 (195)	153 (314)	252 (548)	482 (954)
Single-cell HeLa	557 (853)	1230 (1902)	756 (1024)	1156 (1638)	1481 (2093)	2046 (2591)

The total nonredundant protein count in parentheses, and average proteins per run are outside parentheses.

“MBR+” and “MBR−” indicate that the analysis was performed with and without match-between-runs (MBR), respectively.

Overall, our results above suggest that application of the MBR strategy with no FDR control to sparse datasets, such as single-cell FAIMS data, may result in a high rate of false transfers. IonQuant, with its ability to estimate FDR, provides the users a way to control the rate of false transfers by applying an FDR threshold of their choice. This dataset also invites a discussion regarding a reasonable FDR threshold to apply in different scenarios. In a typical whole cell lysate data, the saturation in the number of quantified proteins is clearly reached at a small FDR threshold (*e.g.*, around 1% FDR in Fig. 2). In such datasets, applying a more lenient FDR threshold is likely to reduce the overall quantification accuracy with no noticeable improvement in the number of quantified proteins. Single-cell datasets, on the other hand, are naturally sparser, with more peptides and proteins that can be transferred from other single-cell runs and especially from the “library” runs (*i.e.*, from boosting samples containing a higher number of cells). In such cases, using a more lenient (*e.g.*, 2%) MBR FDR threshold may be considered, provided that downstream data analysis tools (*e.g.*, for pathway-level analysis) are sufficiently robust toward quantification errors (48).

### Speed of Indexing-Based MBR in IonQuant

Finally, we compared the computational time required by IonQuant (version 1.5.5) and MaxQuant (version 1.6.14.0), both with MBR enabled. The HeLa dataset (timsTOF Pro) and the two-organism dataset from (Orbitrap Fusion Lumos) were used, comprising four and 40 LC-MS files, respectively (Experimental Procedures). For MaxQuant, only jobs related to quantification and MBR were counted (supplemental Tables S10 and S11). Table 5 displays the run time of these tools in minutes. IonQuant is approximately 19 or 38 times faster than MaxQuant in analyzing the data with and without ion mobility, respectively. The reason that IonQuant exhibits a smaller gain in speed compared with MaxQuant when analyzing the timsTOF Pro data is that most of the IonQuant runtime is spent loading the raw data *via* the vendor-provided library (22).

**Table 5:** Run time comparison (in minutes) of quantification-related tasks using the HeLa dataset (4 timsTOF Pro runs) and the two-organism dataset (40 Orbitrap Fusion Lumos runs)

Tool	HeLa	Two-organism
MaxQuant	699	1056
IonQuant	37	28

## Conclusions

MBR is a commonly used approach to quantify additional peptides and proteins by transferring information across different samples. It largely mitigates the missing value problem of DDA-based label-free quantification, increasing data completeness for improved differential analyses. Peptides are transferred from one run to the other by aligning retention time and ion mobility (if applicable). Owing to the dynamic range and complexity of proteomic samples, low signal-to-noise ratios and co-isolation interference can result in incorrectly transferred ions. To our knowledge, there was previously no method to control the rate of false transfers in DDA-based MBR in practical settings. To address this issue, we have described a method to estimate and control the FDR for MBR with the help of mixture modeling and the target-decoy concept. We implemented MBR with FDR control in our quantification tool, IonQuant. Our experiments and comparisons with a frequently used tool MaxQuant showed that IonQuant allowed fewer false positive transfers while maintaining high sensitivity. We also highlight the importance of FDR control when MBR is applied to sparse datasets such as those from single-cell FAIMS proteomics experiments. Furthermore, by way of advanced indexing technology, IonQuant performs MBR with unmatched speed, making it well-suited even for analysis of large-scale datasets.

## Data Availability

The two-organism data was published by Lim *et al.* (18) and can be found at the ProteomeXchange Consortium website (36) with identifier PXD014415. The HeLa cell lysate data were published by Meier *et al.* (23) and can be found at the ProteomeXchange Consortium website with the identifier PXD010012. The three-organism data were published by Prianichnikov *et al.* (24) and can be found at the ProteomeXchange Consortium website with identifier PXD014777. The single-cell data were published by Williams *et al.* (25) and can be found at the ProteomeXchange Consortium website with identifier MSV000085230. MSFragger and IonQuant programs were developed in the cross-platform Java language and can be accessed at http://msfragger.nesvilab.org/ and https://ionquant.nesvilab.org/. Peptide list can be accessed at https://dx.doi.org/10.5281/zenodo.4574598.

## Supplemental data

This article contains supplemental data.

## Conflict of interest

The authors declare no competing financial interests.
