# Supplementary material for: IonQuant Enables Accurate and Sensitive Label-Free Quantification With FDR-Controlled Match-Between-Runs
Source: Mol Cell Proteomics. 2021 Apr 2;20:100077. doi: 10.1016/j.mcpro.2021.100077 (PMC8131922; doi:10.1016/j.mcpro.2021.100077)
Supplement: Supplemental Figures S1–S3 [file mmc1.pdf]

Supplemental Figures for

"IonQuant enables accurate and sensitive label-free quantification with FDR-controlled match-between-runs"

Fengchao Yu[1], Sarah E. Haynes[1], Alexey I. Nesvizhskii[1,2]*

[1]Department of Pathology, University of Michigan, Ann Arbor, Michigan, USA

[2]Department of Computational Medicine and Bioinformatics, University of Michigan, Ann Arbor, Michigan, USA

* Correspondence to A.I.N. (nesvi@med.umich.edu)

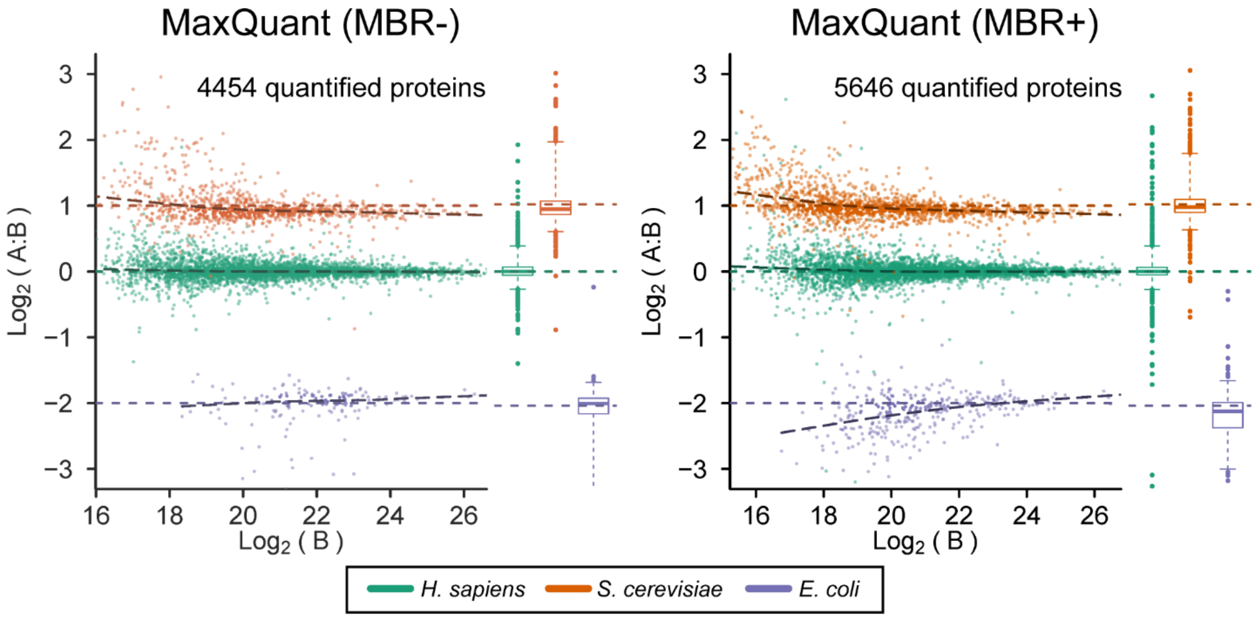

Figure S1. Ground-truth protein quantification results from MaxQuant (version 1.6.14.0) with parameters similar to those used in MSFragger and IonQuant. “MBR+” and “MBR-” indicate the analysis was performed with and without match-between-runs (MBR), respectively. *S. cerevisiae* proteins are shown in orange, *H. sapiens* in green, and *E. coli* in purple. The true ratios of condition A over condition B are 2:1 (*S. cerevisiae*), 1:1 (*H. sapiens*), and 1:4 (*E. coli*). The horizontal colored dashed lines (orange, green, and purple) indicate the true ratios. The black dashed lines are fitted curves from observed ratios. Box plots of the intensities are shown to the right of each scatter plot panel.

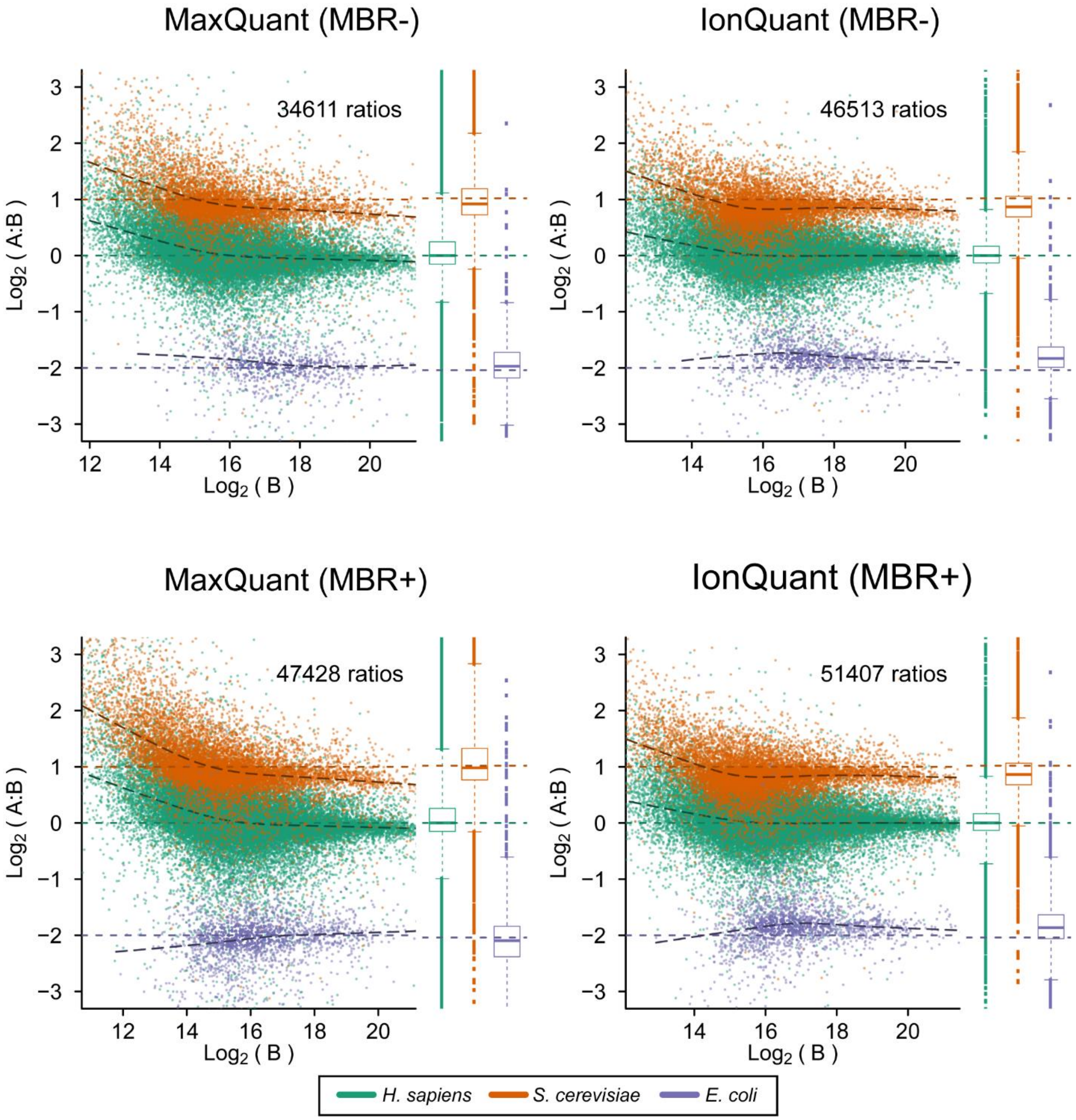

Figure S2. Ground-truth ion quantification results from MaxQuant (version 1.6.14.0) and IonQuant from a mixture of three different proteomes. “MBR+” and “MBR-” indicate that the analysis was performed with and without match-between-runs (MBR), respectively. *S. cerevisiae* proteins are shown in orange, *H. sapiens* in green, and *E. coli* in purple. The known ratios of condition A over condition B are 2:1 (*S. cerevisiae*), 1:1 (*H. sapiens*), and 1:4 (*E. coli*). The horizontal colored dashed lines (orange, green, and purple) indicate the true ratios. The black dashed lines are fitted curves from observed ratios. Box plots of the intensities are shown to the right of each scatter plot panel. The intensities from both tools are not normalized.

	File	Exists	Size	Data format	Parameter group	Experiment	Fraction	PTM	Reference channels
1	F:\data\PXD022791\split\FAIMS_2CV_OTIT_HCD_300ITMS2_Blank_1_-55.mzXML	True	213.8 MB	MzXml file	Group 0	blank_1	1	False	
2	F:\data\PXD022791\split\FAIMS_2CV_OTIT_HCD_300ITMS2_Blank_1_-70.mzXML	True	117.2 MB	MzXml file	Group 0	blank_1	3	False	
3	F:\data\PXD022791\split\FAIMS_2CV_OTIT_HCD_300ITMS2_Blank_2_-55.mzXML	True	176.2 MB	MzXml file	Group 0	blank_2	1	False	
4	F:\data\PXD022791\split\FAIMS_2CV_OTIT_HCD_300ITMS2_Blank_2_-70.mzXML	True	96.2 MB	MzXml file	Group 0	blank_2	3	False	
5	F:\data\PXD022791\split\FAIMS_2CV_OTIT_HCD_300ITMS2_Blank_3_-55.mzXML	True	163.9 MB	MzXml file	Group 0	blank_3	1	False	
6	F:\data\PXD022791\split\FAIMS_2CV_OTIT_HCD_300ITMS2_Blank_3_-70.mzXML	True	77.4 MB	MzXml file	Group 0	blank_3	3	False	
7	F:\data\PXD022791\split\FAIMS_2CV_OTIT_HCD_300ITMS2_Single_HeLa_1_-55.mzXML	True	286.8 MB	MzXml file	Group 0	single_hela_1	1	False	
8	F:\data\PXD022791\split\FAIMS_2CV_OTIT_HCD_300ITMS2_Single_HeLa_1_-70.mzXML	True	171.1 MB	MzXml file	Group 0	single_hela_1	3	False	
9	F:\data\PXD022791\split\FAIMS_2CV_OTIT_HCD_300ITMS2_Single_HeLa_2_-55.mzXML	True	309.2 MB	MzXml file	Group 0	single_hela_2	1	False	
10	F:\data\PXD022791\split\FAIMS_2CV_OTIT_HCD_300ITMS2_Single_HeLa_2_-70.mzXML	True	203.2 MB	MzXml file	Group 0	single_hela_2	3	False	
11	F:\data\PXD022791\split\FAIMS_2CV_OTIT_HCD_300ITMS2_Single_HeLa_3_-55.mzXML	True	305.7 MB	MzXml file	Group 0	single_hela_3	1	False	
12	F:\data\PXD022791\split\FAIMS_2CV_OTIT_HCD_300ITMS2_Single_HeLa_3_-70.mzXML	True	192.3 MB	MzXml file	Group 0	single_hela_3	3	False	
13	F:\data\PXD022791\split\FAIMS_2CV_OTIT_HCD_300ITMS2_100cell_1_-55.mzXML	True	430.5 MB	MzXml file	Group 0	library	1	False	
14	F:\data\PXD022791\split\FAIMS_2CV_OTIT_HCD_300ITMS2_100cell_1_-70.mzXML	True	289.9 MB	MzXml file	Group 0	library	3	False	
15	F:\data\PXD022791\split\FAIMS_2CV_OTIT_HCD_300ITMS2_100cell_2_-55.mzXML	True	408.4 MB	MzXml file	Group 0	library	1	False	
16	F:\data\PXD022791\split\FAIMS_2CV_OTIT_HCD_300ITMS2_100cell_2_-70.mzXML	True	284.3 MB	MzXml file	Group 0	library	3	False	
17	F:\data\PXD022791\split\FAIMS_2CV_OTIT_HCD_300ITMS2_100cell_3_-55.mzXML	True	399.6 MB	MzXml file	Group 0	library	1	False	
18	F:\data\PXD022791\split\FAIMS_2CV_OTIT_HCD_300ITMS2_100cell_3_-70.mzXML	True	270.8 MB	MzXml file	Group 0	library	3	False	

Figure S3. A screenshot of the MaxQuant data annotation page for the single-cell FAIMS data. The files with compensation voltage equal to -55 V was set to fraction 1, and the files with compensation voltage equal to -70 V was set to fraction 3. Files from the same run were set to the same experiment.
